# Tautomycin and enzalutamide combination yields synergistic effects on castration-resistant prostate cancer

**DOI:** 10.1038/s41420-022-01257-1

**Published:** 2022-11-29

**Authors:** Mayao Luo, Yifan Zhang, Zhuofan Xu, Chenwei Wu, Yuedian Ye, Rui Liu, Shidong Lv, Qiang Wei

**Affiliations:** 1 Department of Urology, Nanfang Hospital, Southern Medical University, Guangzhou, Guangdong 510515 China; 2 School of Basic Medical Sciences, Southern Medical University, Guangzhou, Guangdong 510515 China

**Keywords:** Cancer therapeutic resistance, Target validation

## Abstract

The androgen receptor (AR) plays an essential role in prostate cancer progression and is a key target for prostate cancer treatment. However, patients with prostate cancer undergoing androgen deprivation therapy eventually experience biochemical relapse, with hormone-sensitive prostate cancer progressing into castration-resistant prostate cancer (CRPC). The widespread application of secondary antiandrogens, such as enzalutamide, indicates that targeting AR remains the most efficient method for CRPC treatment. Unfortunately, neither can block AR signaling thoroughly, leading to AR reactivation within several months. Here, we report an approach for suppressing reactivated AR signaling in the CRPC stage. A combination of the protein phosphatase 1 subunit α (PP1α)-specific inhibitor tautomycin and enzalutamide synergistically inhibited cell proliferation and AR signaling in LNCaP and C4-2 cells, as well as in AR variant-positive 22RV1 cells. Our results revealed that enzalutamide competed with residual androgens in CRPC, enhancing tautomycin-mediated AR degradation. In addition, the remaining competitive inhibitory role of enzalutamide on AR facilitated tautomycin-induced AR degradation in 22RV1 cells, further decreasing ARv7 levels via a full-length AR/ARv7 interaction. Taken together, our findings suggest that the combination of tautomycin and enzalutamide could achieve a more comprehensive inhibition of AR signaling in CRPC. AR degraders combined with AR antagonists may represent a new therapeutic strategy for CRPC.

## Introduction

Prostate cancer is the most common cancer and the second leading cause of cancer-related deaths in Western men [1]. For high-risk localized disease or metastasis, androgen deprivation therapy (ADT) by chemical or surgical castration is the basic treatment, in addition to radical prostatectomy and radiotherapy. Unfortunately, patients who undergo ADT often eventually progress to a lethal stage of castration-resistant prostate cancer (CRPC). At this stage, the patients do not respond to ADT, and the median survival time is only 1–2 years. However, evidence indicates that the androgen receptor (AR)-signaling pathway is still pivotal for CRPC. Increased prostate-specific antigen (PSA) levels are detected in most patients with CRPC [2, 3]. Thus, more efforts are required to explore new approaches for blocking AR signaling.

AR is a member of the steroid receptor family of transcription factors that share structurally conserved domains, consisting of an N-terminal domain (NTD), a DNA-binding domain, a ligand-binding domain (LBD), and a hinge region containing a nuclear localization sequence [4]. The importance of AR in prostate cancer development and progression makes it a major target for prostate cancer treatment [5]. ADT uses approaches that target LBD, including directly binding LBD by AR competitive inhibitors, or reducing the level of androgens via LHRH/GnRH analogs and CYP17 inhibitors [6–8]. However, in the CRPC stage, AR is reactive due to alterations in expression, structure, and stability [2, 3]. Copy-number amplification or overexpression and gain-of-function mutations lead to AR activation by other steroids or even antiandrogens [9–11]. The presence of constitutively active splice variants causes the AR to lose the domain responsible for binding to its competitive inhibitors [12, 13]. Increased stability contributes to AR nuclear accumulation, which increases its sensitivity to low-level androgens [14]. These alterations and other mechanisms, such as overexpression of coactivators or activation of other ligand-independent roles [15], would maintain AR transactivation despite castrate levels of androgen and resist current antiandrogen treatment.

Recently, some attempts have been made to further block the reactivated AR in CRPC. EPI-001 and its analogs are small molecular inhibitors of the NTD, which could overcome the shortcomings of current therapies targeting the LBD [16]. Selective AR degraders, such as AZD3514 [17] or ASC-J9 [18], and proteolysis targeting chimeras, such as ARV-771 [19] or ARCC-4 [20], are designed to immediately reduce AR protein expression. Moreover, ADT induces susceptibility in prostate cancer cells, rendering them amenable to synergistic treatment. Several clinical studies have shown that combination therapy leads to better outcomes than single-drug therapy [21]. Further clinical trials are ongoing, such as with the NTD inhibitor EPI-7386, the LBD inhibitors enzalutamide (NCT05075577) and CYP17A1, and the proteolysis targeting chimera-type inhibitor abiraterone ARV-110 (NCT05177042). These combinations could overcome the limitations of single drugs and represent a new strategy against clinically reactivated AR.

Here, we explored a newly identified drug combination of enzalutamide and a protein phosphatase 1 subunit α (PP1α) inhibitor tautomycin, which contributed to the increased stability and nuclear accumulation of AR in CRPC. These two drugs synergically inhibited cell proliferation, especially for AR variant-positive cells. The enzalutamide could significantly enhance the tautomycin-mediated degradation of AR and AR-v7 via the ubiquitin–proteasome pathway. These findings suggest the potential of a combination of enzalutamide and tautomycin to develop novel therapeutics for CRPC.

## Results

### A combination of tautomycin and enzalutamide synergistically inhibits cell proliferation

**Fig. 1:**
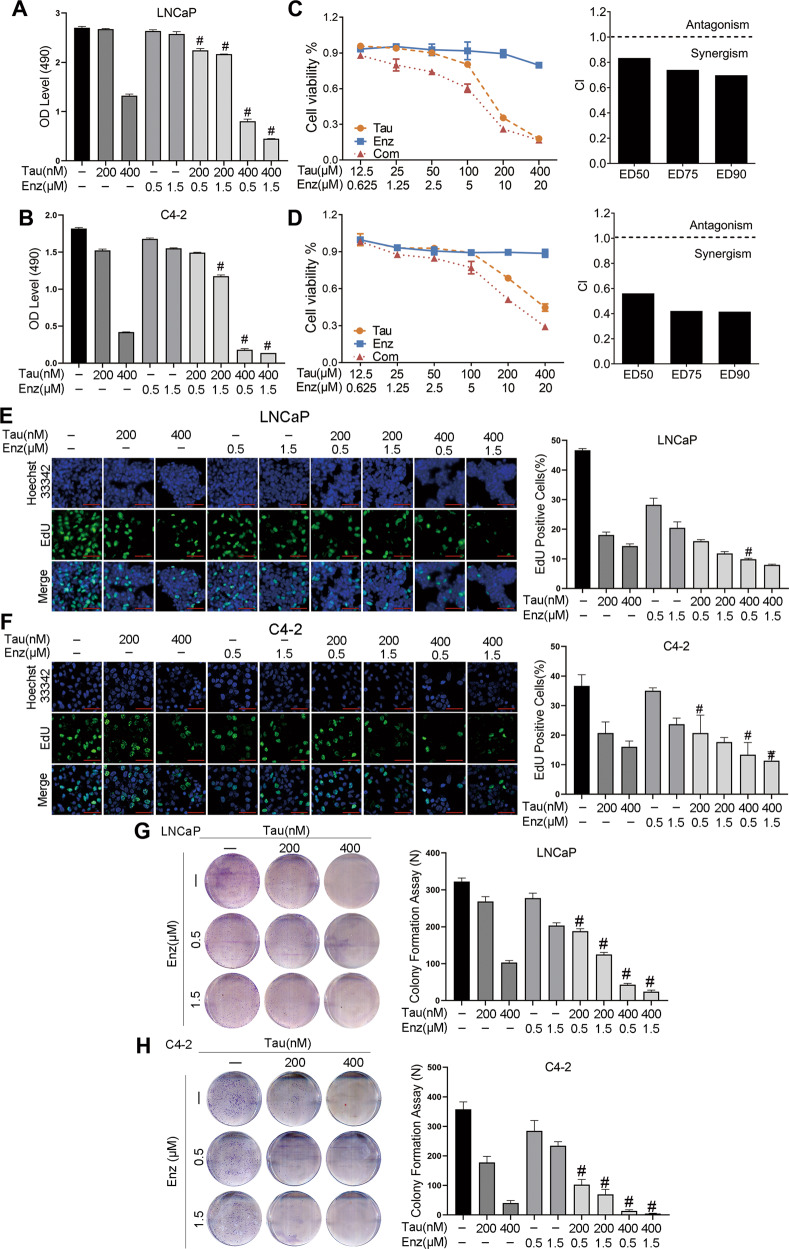
Combination of tautomycin and enzalutamide synergistically inhibited prostate cancer cell viability. **A**, **B** The cell viability of LNCaP (**A**) and C4-2 (**B**) were measured using MTT assay after being treated with indicated drugs and 1 nM DHT for 48 h. **C**, **D** The synergist effect of tautomycin and enzalutamide was calculated with the Chou-Talalay method as described previously. The LNCaP (**C**) and C4-2 (**D**) cells were treated with a gradient dose of tautomycin and enzalutamide individually or in combination, and the cell viability was detected using MTT assay after 48 h. Represented images of EdU incorporation in LNCaP (**E**) and C4-2 (**F**) cells treated with tautomycin and enzalutamide in the presence of 1 nM DHT. Nuclei were stained with Hoechst 33342 (scale bars: 50 μm). **G**, **H** Represented images of colony formation assay in LNCaP (**G**) and C4-2 (**H**) cells. ^#^Synergy by bliss-independent analysis.

To explore the effect of the tautomycin and enzalutamide combination, an MTT assay was performed to detect cell viability in LNCaP and C4-2 cells. As shown in Fig. 1A, B, both tautomycin and enzalutamide dose-dependently reduced cell viability. In the combination treatment group, we observed the most dramatic inhibition of cell proliferation compared to the single-drug group. The combination index (CI) developed by Chou-Talalay was used to evaluate the synergistic effect of two different drugs. We calculated the CI of tautomycin and enzalutamide in LNCaP and C4-2 cells. The results showed that the CI values were lower than 1, indicating synergism between the two drugs (Fig. 1C, D). Two additional cell proliferation assays were performed. In line with the MTT assay results, the EdU and colony formation assays supported the combination of tautomycin and enzalutamide, which showed the most dramatic inhibition of prostate cancer cell growth (Fig. 1E–H). Overall, these findings provide compelling evidence of a synergistic effect between tautomycin and enzalutamide on cell growth in prostate cancer.

### A combination of tautomycin and enzalutamide synergistically inhibits AR signaling activity

**Fig. 2:**
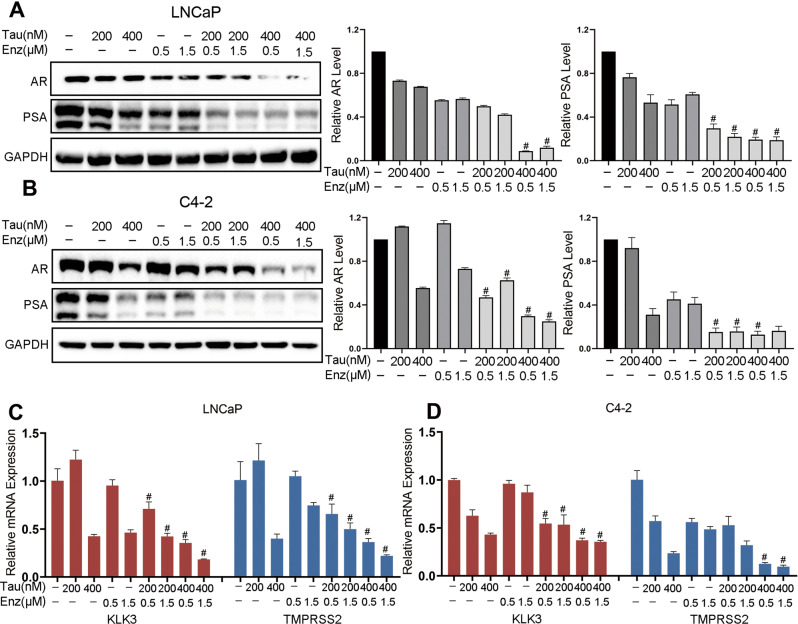
Combining tautomycin and enzalutamide synergistically decreased AR protein level and inhibited AR downstream genes expression. **A**, **B** The protein levels of AR, PSA, and GAPDH were detected using Western blot in LNCaP (**A**) and C4-2 (**B**) cells, and quantitative levels are shown on the left. **C**, **D** The mRNA levels of KLK3 and TMPRSS2 were explored using qRT-PCR in LNCaP (**C**) and C4-2 (**D**) cells with indicated treatment. ^#^Synergy by bliss-independent analysis.

The mechanism underlying the inhibition of proliferation by tautomycin and enzalutamide mainly relies on targeting and decreasing the AR signaling activity. Therefore, to investigate the synergistic effect of tautomycin and enzalutamide, we evaluated AR signaling using Western blotting and real-time quantitative reverse transcription PCR (RT-qPCR). As shown in Fig. 2A, B, we first examined the protein levels of AR and PSA in LNCaP and C4-2 cells. With 1 nM DHT treatment, both tautomycin and enzalutamide showed limited effects on AR signaling. We observed only a slight decrease in PSA levels. However, the combination treatment yielded the most marked downregulation. In addition to protein levels, mRNA levels of AR downstream targets were measured. Consistently, the combination of tautomycin and enzalutamide maximally reduced AR signaling compared to a single drug (Fig. 2C, D). Taken together, the combination of tautomycin and enzalutamide synergistically inhibited AR signaling activity, thereby reducing cancer cell proliferation.

### Enzalutamide enhances tautomycin-induced AR degradation

**Fig. 3:**
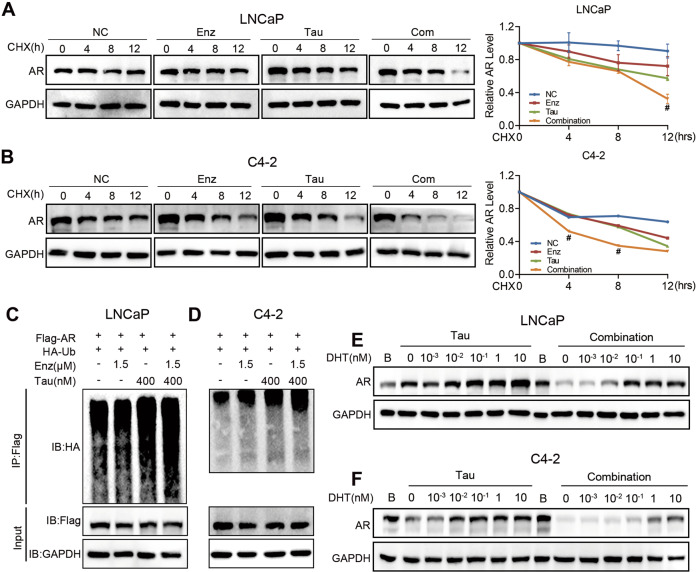
Enzalutamide amplified the degradation effect of tautomycin. **A**, **B** The AR protein half-life of LNCaP (**A**) and C4-2 (**B**) cells were evaluated using Western blot in the presence of 100 μg/mL cycloheximide after cells were treated with 400 nM tautomycin and 1.5 μM enzalutamide for 4, 8, and 12 h. Bands were quantified using ImageJ and shown in the right panel. **C**, **D** The protein ubiquitin level of AR was tested using co-immunoprecipitation using anti-Flag magnetic beads. The LNCaP (**C**) and C4-2 (**D**) cells were transfected with Flag-AR and HA-Ubiquitin plasmid and then treated with indicated concentrations of tautomycin and enzalutamide in the presence of 10 μM MG132 for 48 h. **E**, **F** The AR level was detected using Western blot in LNCaP (**E**) and C4-2 (**F**) cells after treatment with gradient DHT and 400 nM tautomycin in the presence or absence of 1.5 μM enzalutamide. ^#^Synergy by bliss-independent analysis.

PP1α reportedly regulates AR protein stability via MDM2, an E3 ubiquitin ligase responsible for the intracellular degradation of AR [22]. As shown in Fig. 2A, B, the protein level of AR was lower in the combination treatment group than in the tautomycin alone group. This suggests that enzalutamide may increase the effect of tautomycin on AR degradation. To test this hypothesis, the half-life of AR was detected with cycloheximide treatment to inhibit protein synthesis in LNCaP and C4-2 cells. We found that AR stability was decreased in the tautomycin single-drug treatment group, consistent with our previous findings [14]. Notably, in the presence of enzalutamide, the effect of tautomycin was amplified, and the half-life of AR was shorter in the combination group than in other groups (Fig. 3A, B). To confirm this observation, we detected AR-ubiquitin levels via co-immunoprecipitation. Expectedly, the highest level of AR ubiquitination was identified in the combination group, substantiating the pivotal role of enzalutamide in tautomycin-induced AR degradation (Fig. 3C, D). In the half-life and ubiquitin assays, we noticed that, except for tautomycin, enzalutamide also reduced AR stability (Fig. 3A–D). This might be because cells were cultured in a regular medium containing castrated-level androgens. As a competitive inhibitor, enzalutamide competes with androgen for binding to AR, which decreases its stability. Thus, the mechanism underlying the synergistic effect between tautomycin and enzalutamide might be because enzalutamide treatment keeps AR unbound to the ligand, rendering it more easily accessible to AR degraders, such as tautomycin. To test this hypothesis, we cultured cells with a gradient concentration of DHT and treated them with tautomycin in the presence or absence of enzalutamide. The results are shown in Fig. 3E, F. With increased DHT levels, the effect of tautomycin was attenuated due to increased AR ligand–receptor interactions. However, after the combination with enzalutamide, the influence of DHT was markedly decreased. These findings suggest that enzalutamide prevents androgen binding to AR, enhancing the effect of tautomycin.

### A combination of tautomycin and enzalutamide inhibits AR variant-positive cell proliferation

**Fig. 4:**
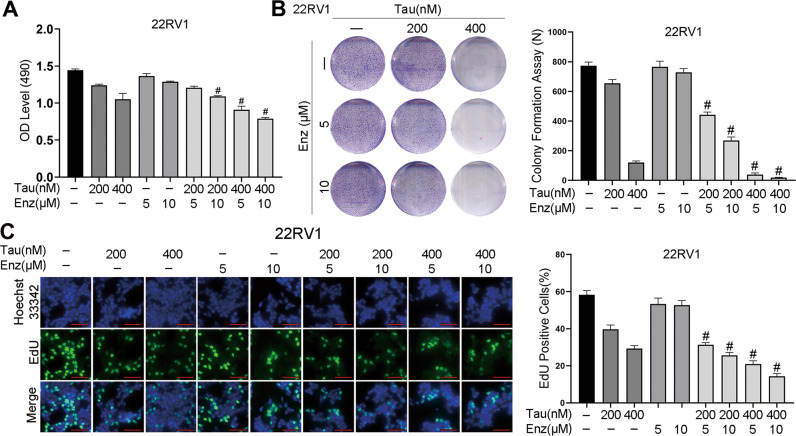
A combination of tautomycin and enzalutamide inhibited ARV-positive 22RV1 cells proliferation. **A** The cell viability of 22RV1 was detected using MTT assay after treatment with indicated drugs. **B** Representative images of colony formation assay in 22RV1 cells treated with tautomycin and enzalutamide. **C** Representative images of EdU incorporation in 22RV1 cells treated with tautomycin and enzalutamide. Nuclei were stained with Hoechst 33342 (scale bars: 50 μm). ^#^Synergy by bliss-independent analysis.

The increased AR degradation after tautomycin and enzalutamide combination treatment overcame AR reactivation such as AR overexpression or stability evaluation. We further aimed to identify whether this combination could prevent AR reactivation caused by structural alterations. 22RV1 cells are known to express both full-length AR (FL-AR) and AR variants (ARVs) that are enzalutamide-resistant. The MTT, EdU, and colony formation assays were repeated. As shown in Fig. 4A, enzalutamide had a limited effect on 22RV1 cell viability. Unlike enzalutamide, ARVs did not affect the inhibition of tautomycin, and the cell viability in the tautomycin-treated group was significantly decreased. This was due to the tautomycin-targeted AR promoting the activity of MDM2, which binds to AR at the NTD. Interestingly, the combination treatment group showed the most remarkable inhibition of proliferation across all four groups. The colony formation assay (Fig. 4B) and EdU assay (Fig. 4C) further supported the results of the MTT assay. Although enzalutamide did not reduce cell proliferation, it did potentiate the effect of tautomycin. Overall, the combination of tautomycin and enzalutamide could overcome the antiandrogen resistance of ARVs.

### Enzalutamide binds to AR in ARV-positive cells, facilitating tautomycin-mediated AR degradation

**Fig. 5:**
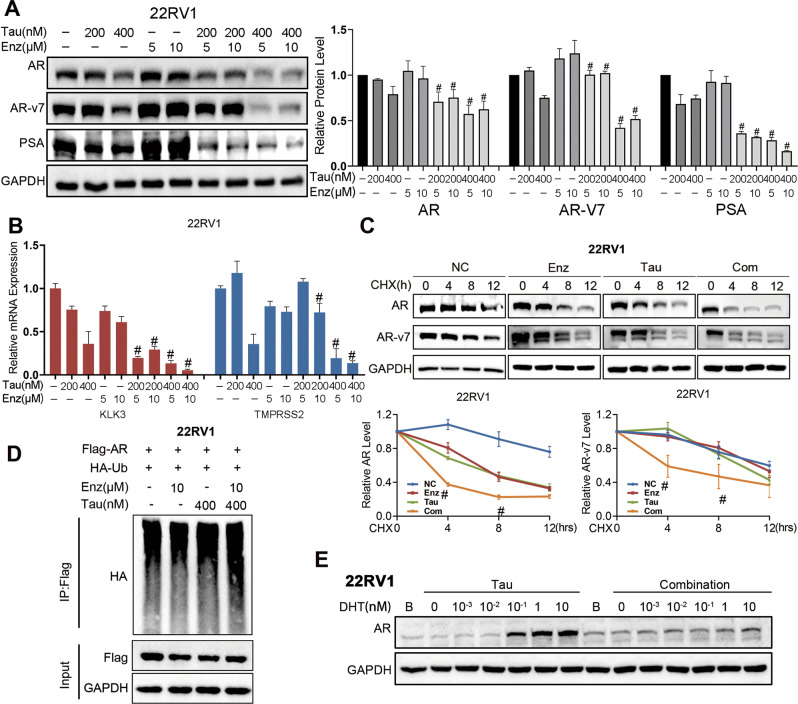
Enzalutamide facilitated the degradation activity of tautomycin in 22RV1 cells. **A** The protein levels of AR, AR-v7, PSA, and GAPDH were detected by Western blot in 22RV1 cells treated with indicated drugs and 1 nM DHT for 48 h. **B** The mRNA levels of KLK3 and TMPRSS2 were examined using qRT-PCR in 22RV1 cells with 1 nM DHT and indicated drug treatment for 48 h. **C** The half-life of AR and AR-v7 protein was explored in 22RV1 using Western blot in the presence of cycloheximide after 400 nM tautomycin or 10 μM enzalutamide treatment for 4, 8, and 12 h. **D** The ubiquitin level of AR was evaluated using co-immunoprecipitation in 22RV1 cells in the presence of 10 μM MG132 for 48 h. **E** The AR level was detected using Western blot in 22RV1 cells after treatment with gradient DHT and 400 nM tautomycin in the presence or absence of 1.5 μM enzalutamide. ^#^Synergy by bliss-independent analysis.

**Fig. 6:**
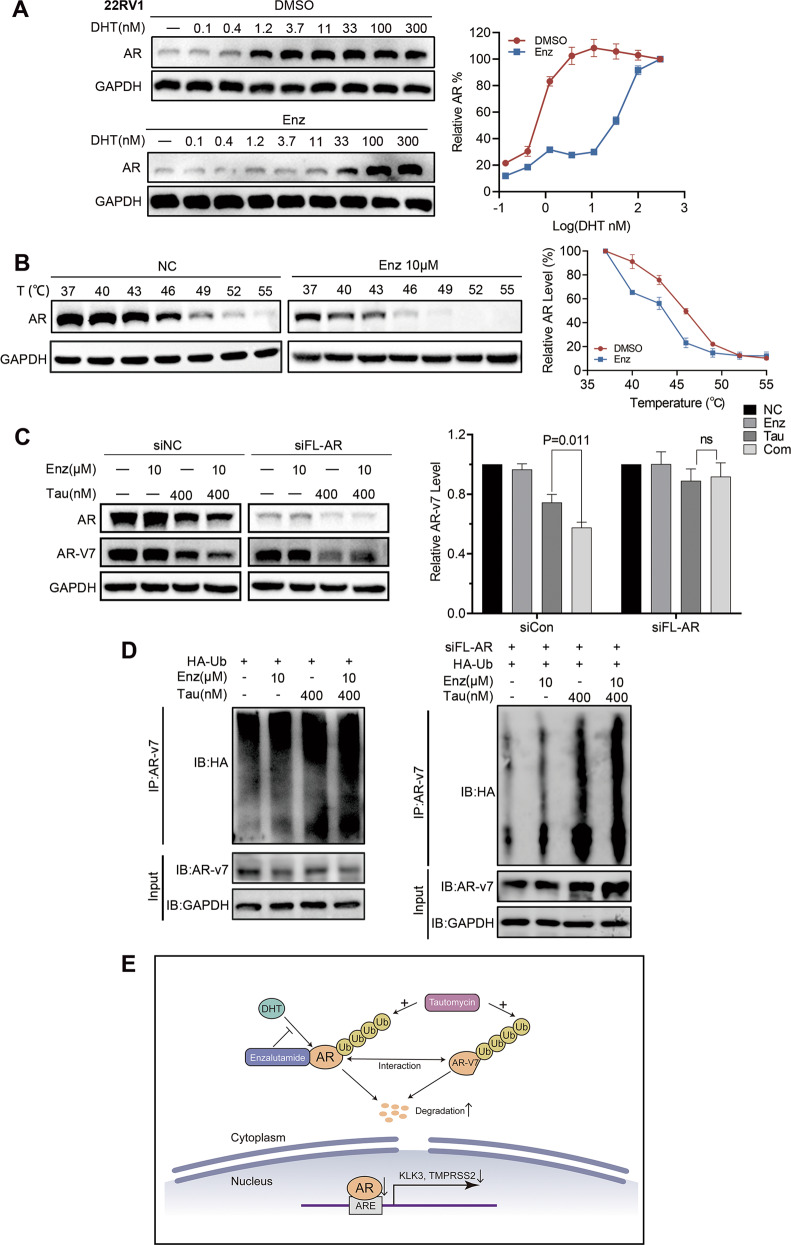
Enzalutamide binds to AR in 22RV1 cells, influencing the interaction between AR and ARv7. **A** ITDRF_CETSA_ experiments performed for DHT in the presence of 50 μM enzalutamide in 22RV1 cells for 1 h. **B** CETSA experiments were performed following indicated heat shocks in 22RV1 cells after treating with 10 μM enzalutamide for 48 h. **C** The protein levels of ARv7 were analyzed using Western blot in 22RV1 cells transfected with FL-AR siRNA and treated with indicated concentrations of tautomycin and enzalutamide for 48 h. **D** The ubiquitin level of ARv7 was evaluated using co-immunoprecipitation in 22RV1 cells in the presence of 10 μM MG132 for 48 h with or without knockdown of full-length AR. **E** A schematic model for the mechanism through which a combination of tautomycin and enzalutamide inhibits the proliferation of prostate cancer. *P* < 0.05 was accepted as significant, ns as not significant.

To assess the AR signaling alteration after tautomycin and enzalutamide combination treatment, we next detected the protein and mRNA levels of AR, ARv7, and their downstream targets. Tautomycin decreased the protein levels of both FL-AR and ARVs, while enzalutamide acted as an enhancer of tautomycin effects (Fig. 5A). With the reduced protein level, the AR signaling activity decreased in the tautomycin and combination treatment groups, while enzalutamide dramatically promoted the activity of tautomycin (Fig. 5A, B). Furthermore, the half-lives of AR and AR-v7 proteins were detected in 22RV1 cells. As expected, tautomycin promoted the degradation of both FL-AR and ARVs. Meanwhile, we observed the lowest AR and ARv7 stability in the combination treatment group (Fig. 5C). Similarly, the AR-ubiquitin level was evaluated in 22RV1 cells by co-immunoprecipitation, and the combination treatment significantly increased the level of ubiquitinated AR (Fig. 5D). Reduced AR stability following enzalutamide treatment was also observed in 22RV1 cells cultured in a regular medium. Therefore, a gradient DHT assay was performed. The result was the same as that in LNCaP and C4-2 cells; enzalutamide treatment competed with DHT and increased tautomycin-mediated AR degradation. These findings indicate that although enzalutamide does not inhibit proliferation and AR signaling activity due to the presence of ARVs, it could still interact with AR and compete for androgen binding, sensitizing cells to tautomycin. This hypothesis was supported by CETSA, which was used to detect direct binding of the ligand and receptor. After enzalutamide treatment, DHT binding affinity was remarkably decreased in 22RV1 cells (Fig. 6A), indicating that enzalutamide still acted as a competitive antagonist in 22RV1 cells. In addition, we performed a long-term CETSA to detect the AR protein interaction status (PRINTS) by treating cells with enzalutamide for 48 h. We found that enzalutamide could dramatically decrease the thermal stability of AR in 22RV1 cells (Fig. 6B). Thus, the binding of enzalutamide and AR would influence the status of AR even in drug-resistant cell lines.

In Fig. 5, our results showed that the combination of tautomycin and enzalutamide not only decreased the level of FL-AR but also targeted ARVs. Previous research has reported an interaction between FL-AR and ARVs in ARV-positive prostate cells and that the transcription of ARVs downstream targets is required for FL-AR [23]. Therefore, we hypothesized that the synergistic effect on ARVs relies on FL-AR. Thus, we knocked down FL-AR expression using siRNA. As shown in Fig. 6C, tautomycin decreased ARv7 expression in 22VR1 cells, with or without FL-AR. However, enzalutamide failed to facilitate the degradation of tautomycin in the FL-AR silenced group. The same results were also identified in the ARv7 ubiquitin assay, in absence of FL-AR, we didn’t observe any increase of ARv7 ubiquitination in combination group compared to the tautomycin treatment group (Fig. 6D). These results indicated that the combination effect of enzalutamide and tautomycin on ARv7 might be mediated through FL-AR/ARv7 heterodimerization.

## Discussion

Prostate cancer is an endocrine-related cancer, and the AR signaling pathway, which participates in the entire disease process, is indispensable for cancer growth and distant metastasis [24]. Thus, using drugs to target the AR pathway is a front-line treatment for patients with prostate cancer. Contemporary ADT for prostate cancer typically involves chemical castration through the chronic use of GnRH agonists or antagonists, which lowers testosterone levels by stably suppressing androgen secretion from the testes [3]. In addition to chemical castration, a competitive AR antagonist is routinely used to eliminate androgens in the prostate. This incorporation has been termed combined androgen blockade (CAB) and is now in widespread clinical use. However, although CAB could decrease androgen activity, tumor cells still deploy several strategies to escape from CAB. For instance, ADT only targets androgen-form testes, while adrenal androgen, intraprostatic testosterone, and DHT synthesis also exist. With the increased sensitivity of receptors due to overexpression, mutation, or nuclear accumulation, AR signaling is reactivated, and the disease progresses to CRPC [10, 25, 26]. In this stage, secondary antiandrogens, such as abiraterone and enzalutamide, are applied to impede AR reactivation by reducing intraprostatic testosterone synthesis or competing with ligand–receptor binding. However, each of these approaches targets the LBD of AR and has a narrow therapeutic index. Cancer cells develop drug resistance within a short time [27]. Therefore, a more thorough AR blockade method is urgently required. Here, we provide a new combination strategy, comprising enzalutamide and AR degrader, tautomycin, to maximize AR inhibition in CRPC (Fig. 6D). Our results showed that tautomycin and enzalutamide synergistically inhibited cell proliferation in LNCaP and C4-2 cells and exerted an ideal effect on AR variant-positive 22RV1 cells. Consistently, AR signaling was significantly decreased after treatment with tautomycin and enzalutamide in LNCaP, C4-2, and 22RV1 cells. These findings provide a valuable strategy to block AR reactivation in CRPC continually. Moreover, as enzalutamide and tautomycin target AR by different mechanisms, this might contribute to reducing the potential for developing resistance.

Tautomycin is a PP1α inhibitor. As a protein phosphatase, PP1α negatively regulates E3 ligase MDM2 and SKP2 activity by decreasing their phosphorylation [28]. Accumulating evidence confirms that turnover of AR occurs through the ubiquitin–proteasome pathway [14]. Both MDM2 and SKP2 are major executors of AR degradation. Moreover, the interaction between the AR and MDM2 or SKP2 occurs at the NTD site. Thus, PP1α is not only an important regulator of AR degradation but also regulates ARV turnover [29]. In our previous research, we found that in CRPC cells, the level of PP1α was expectedly increased, which downregulated the activity of MDM2, contributing to the high stability of AR. With the close relationship between AR stability and nuclear accumulation, PP1α is also considered to promote the transition of AR from cytoplasmic distribution to nuclear accumulation along with CRPC progression. These findings indicate that PP1α is a promising candidate for CRPC, while targeting it could simultaneously reverse the alteration of AR expression, structure, and stability. However, the effect of tautomycin alone is unsatisfactory and easily attenuated by androgens. Nishiyama et al. reported that even in patients who underwent castration, the residual concentration of intraprostatic DHT is approximately 4.6 nM, higher than the DHT we used [30]. Therefore, we combined tautomycin with enzalutamide. Benefiting from the competitive effect of enzalutamide on AR, tautomycin can overcome the influence of residual androgen and target the AR protein more dramatically. Indeed, our results confirmed that this combination could greatly enhance the activity of tautomycin and achieve greater inhibition of AR signaling in CRPC.

Enzalutamide is a secondary antiandrogen developed for the treatment of CRPC. Compared with first-generation antiandrogens, enzalutamide shows approximately 8-fold greater affinity than bicalutamide. Previous studies have reported that enzalutamide could block ligand–receptor binding, nuclear translocation, DNA-binding, and coactivator peptide recruitment, thereby affecting the inhibition efficiency of AR downstream target transcription [31]. However, enzalutamide was developed from a nonsteroidal agonist of AR and still works as a competitive inhibitor by targeting the LBD. Thus, the molecular alterations that led to resistance to bicalutamide might also induce resistance to enzalutamide. With the widespread use of enzalutamide in clinical practice, the development of resistance has been observed in most patients [27, 32]. The CWR22 xenograft-derived 22RV1 cell line, which expresses high levels of AR-V7, is a classical model for enzalutamide resistance [33]. Our results confirmed that enzalutamide did not inhibit either proliferation or AR activity in 22RV1 cells. Intriguingly, although enzalutamide did not show any effect on 22RV1 cells, it could serve as an enhancer that amplified the activity of tautomycin to further downregulate AR signaling. The CETSA was initially developed to investigate drug-target engagement in live cells based on altered protein thermostability [34]. Recent research indicates that alterations in protein thermal stability could also reflect changes in PRINTS [35, 36]. Here, we used two different CETSA to elucidate the role of enzalutamide in 22RV1 cells. The decreased stability and curve shift of AR after long-term enzalutamide incubation treatment revealed that the binding of enzalutamide alters the PRINTS of AR and allows it to be easily targeted by other drugs, including the AR degrader tautomycin. Thus, regardless of sensitivity or resistance, enzalutamide should become a primary choice for antiandrogen treatment in CRPC.

In 22RV1 cells, in addition to the finding that enzalutamide could facilitate tautomycin to further decrease AR signaling, we noticed that enzalutamide also helped block ARVs signaling. Most ARVs are truncations of AR and do not have the LBD; therefore, they are constitutively activated in the absence of androgen or the presence of an AR competitive inhibitor, including enzalutamide. Recently, numerous studies have reported the interaction between AR and ARVs. Xu et al. observed that ARv7 and ARv567es heterodimerize with FL-AR in an androgen-independent manner [23]. Moreover, Watson et al. reported that although some ARVs promote castration resistance, they still rely on FL-AR [37]. These studies confirmed that ARVs are not truly independent from AR but rather rely on each other. We considered that the additional effect of tautomycin in combination was due to enzalutamide impairing the interaction between FL-AR and ARVs. Thus, the combination of enzalutamide and tautomycin is suitable for patients with CRPC with ARVs.

In summary, we provide a new potential strategy for CRPC treatment. Our results revealed that the combination of tautomycin and enzalutamide inhibits prostate cancer cell proliferation by synergistically promoting AR and ARv7 protein degradation via the ubiquitin–proteasome pathway. These findings also raise the possibility of combining AR degraders with AR antagonists to treat CRPC and AR antagonists resistant CRPC.

## Materials and methods

### Cell culture and transfection

The prostate cancer cell lines LNCaP, C4-2, and 22RV1 were purchased from Procell Life Science & Technology Co. Ltd. (Wuhan, China). All the cell lines were cultured in RPMI 1640 medium (Corning) supplemented with 10% FBS (Excell Bio, China) or charcoal-stripped FBS (Biological Industries) and 1% penicillin/streptomycin (Thermo Fisher Scientific) in a CO_2_ incubator at 37 °C. FL-AR siRNA (CGUGCAGCCUAUUGCGAGAUU) was synthesized by GenePharma (Shanghai, China). Hemagglutinin (HA)-ubiquitin was a gift from Edward Yeh (Addgene, plasmid # 18712). The Flag-AR plasmid was synthesized by GeneChem (Shanghai, China). Lipofectamine 3000 Transfection Reagent (Thermo Fisher Scientific) was used to transfect both siRNA and plasmids according to the manufacturer’s instructions. All cell lines were authenticated by STR profiling and tested negative for mycoplasma contamination.

### MTT assay

Cells (1 × 10^4^ cells/well) were seeded in 96-well plates. After 24 h, cells were treated with enzalutamide (MedChemExpress, Shanghai, China) and tautomycin (Wako) at various concentrations. Cells were then grown for 48 h, and cell viability was evaluated using the MTT (Sigma) assay, as previously described [38].

### 5-ethynyl-20-deoxyuridine (EdU) proliferation assay

LNCaP, C4-2, and 22RV1 cells (1 × 10^4^ cells/well) were seeded in 96-well plates. After 24 h, cells were treated with enzalutamide (MedChemExpress) and tautomycin (Wako, Japan) at various concentrations, followed by incubation with 5-ethynyl-20-deoxyuridine (EdU, Ribobio, Guangzhou, China) for 2 h, and processed according to the manufacturer’s protocols. The 1× Apollo reaction cocktail and Hoechst 33342 (5 μg/mL) were used to stain the cells for 30 min and then visualized using a fluorescence microscope. EdU-positive cell density was calculated using ImageJ software (RRID:SCR_003070) according to the presence or absence of nuclear-specific staining (Hoechst 33342) compared with negative controls.

### Colony formation assay

LNCaP, C4-2, or 22RV1 cells were seeded in 6-well plates (3000 cells/well), and after 2 days, they were treated with enzalutamide and tautomycin at various concentrations and then cultured in a cell incubator for another two weeks. The cells were fixed with 4% paraformaldehyde for 10 min. The cells were then stained with 0.5% crystal violet for 20 min. The number of cells was calculated using ImageJ software (RRID:SCR_003070).

### Quantitative real-time PCR

Prostate cancer cells were seeded in 12-well plates and treated with enzalutamide and tautomycin at the indicated concentrations. After 48 h, TRIzol reagent (Invitrogen) was used for RNA extraction, according to the manufacturer’s instructions. Reverse transcription was performed with 1 μg of RNA using HiScript^®^ III All-in-one RT SuperMix (Vazyme Biotech Co. Ltd.). Taq Pro Universal SYBR qPCR Master Mix (Vazyme Biotech Co., Ltd.) was used for real-time quantitative PCR on a QuantStudio™ 6 Flex system (Thermo Fisher Scientific). The data were analyzed using the 2^–ΔΔCt^ method.

### Western blot

Prostate cancer cells were lysed in RIPA buffer containing a proteasome inhibitor cocktail (EDTA-free, 100× in dimethyl sulfoxide, Bimake, China), following the manufacturer’s instructions. The protein concentration was determined using the Pierce™ BCA Protein Assay Kit (23227, Thermo Fisher Scientific). Proteins were mixed with protein loading buffer and boiled at 100 °C for 10 min. From each group, 30 μg of protein were separated using 10% SDS-PAGE and subjected to Western blot analysis. The primary antibodies used in this study were as follows: AR (Santa Cruz Biotechnology Inc., cat. sc-7305, 1:1000), PSA (CST, cat. 5365, 1:1000), DYKDDDDK Tag horseradish peroxidase conjugate (CST, cat. 86861, 1:5000), AR-v7 (Abcam, cat. ab198394, 1:1000), HA Tag (CST, cat. #3724, 1:1000), and GAPDH (Santa Cruz Biotechnology Inc., cat. sc-47724, 1:1000). Full-length western blots are provided in supplementary materials.

### Protein half-life assay

Cells were seeded in 6-well plates and, after 24 h, were treated with cycloheximide (Solarbio, 100 µg/mL), enzalutamide, and tautomycin at the indicated concentrations. RIPA buffer containing a proteasome inhibitor cocktail was used to lyse cells after 0, 4, 8, and 12 h. Then, the protein was subjected to Western blot analysis to evaluate AR or AR-v7 protein levels.

### Co-immunoprecipitation

Prostate cancer cells transfected with HA-ubiquitin and Flag-AR were treated with MG132 (10 µM), enzalutamide, or tautomycin at the indicated concentrations. After 24 h, the cells were lysed, incubated with anti-Flag magnetic beads (Bimake, China), and incubated overnight at 4 °C. The anti-Flag magnetic beads were pelleted, washed three times with immunoprecipitation wash buffer, and then eluted with 1× protein sample loading buffer at 100 °C for 5 min.

### Isothermal dose-response-cellular thermal shift assay (ITDRF_CETSA_)

The cells were seeded into a 100 mm dish, and after they reached 80% confluence, they were digested and divided into separate aliquots and exposed to enzalutamide (50 µM) or dimethyl sulfoxide at a gradient concentration of dihydrotestosterone (DHT, Abmole) for 1 h in a cell incubator. 46 °C for 3 min was used to perform heat shock and the lysed by freeze-thaw cycle three times using liquid nitrogen. The cell lysate was centrifuged at 20,000 × *g* for 20 min, and 5× protein sample loading buffer was added to the supernatant after boiling at 100 °C for 10 min and detected using Western blotting.

### Cellular thermal shift assay (CETSA)

CETSA experiments were performed according to the general CETSA protocol [39]. Briefly, 22RV1 cells were seeded into a 100 mm dish, and once they reached 80% confluence, they were treated with enzalutamide (10 µM) or dimethyl sulfoxide for 48 h. Cells were then harvested and resuspended in phosphate-buffered saline. Treated samples were aliquoted and heated at different temperatures for 3 min in a PCR plate (Veriti thermal cycler, Thermo Scientific). Next, a protease inhibitor cocktail was added before lysing cells by three freeze-thaw cycles using liquid nitrogen and a heat block. The cell lysate was centrifuged at 20,000 × *g* for 20 min. Supernatants were transferred to new tubes, boiled at 100 °C for 10 min, and detected by Western blot.

### Statistical analysis

Statistical analyses were performed using GraphPad Prism (version 8.3.1; for Windows, RRID:SCR_002798). Data are presented as the mean ± SEM from at least three biological replicates. The two-tailed Student’s *t*-test was used for data comparison between the two groups. One-way analysis of variance (ANOVA) was used to compare two or more groups. Two-way ANOVA tests, the bliss-independent model [40], and Chou-Talalay method [41] were used to determine the synergistic effect. The determination of synergy was listed in Table S1.

## Supplementary information

Supplementary materials

supplementary full length western blot

Table S1. Determination of synergy

## Data Availability

The data that support the findings of this study are available from the corresponding author upon reasonable request.
