# Supplementary material for: Tautomycin and enzalutamide combination yields synergistic effects on castration-resistant prostate cancer
Source: Cell Death Discov. 2022 Nov 29;8:471. doi: 10.1038/s41420-022-01257-1 (PMC9708830; doi:10.1038/s41420-022-01257-1)
Supplement: Supplementary file 1 — Supplementary materials [file 41420_2022_1257_MOESM1_ESM.docx]

Supplementary Information

**Tautomycin and enzalutamide combination yields synergistic effects on castration-resistant prostate cancer**

Mayao Luo^1^, Yifan Zhang^1^, Zhuofan Xu^1^, Chenwei Wu^1^, Yuedian Ye^1^, Rui Liu^2*^, Shidong Lv^1*^, Qiang Wei^1*^

^1^Department of Urology, Nanfang Hospital, Southern Medical University, Guangzhou, Guangdong, 510515, China

^2^School of Basic Medical Sciences, Southern Medical University, Guangzhou, Guangdong, 510515, China

Primer sequence for qPCR

| KLK3 F | CAGGTGTAGACCAGAGTGTTTC |
| --- | --- |
| KLK3 R | CTGTGTCCTCAGAGAAATTGAGT |
| TMPRSS2 F | TGCTCCAACTCTGGGATAGA |
| TMPRSS2 R | GGATGAAGTTTGGTCCGTAGAG |
| GAPDH F | CTCCTCACAGTTGCCATGTA |
| GAPDH R | GTTGAGCACAGGGTACTTTATTG |
