# Supplementary material for: Tautomycin and enzalutamide combination yields synergistic effects on castration-resistant prostate cancer
Source: Cell Death Discov. 2022 Nov 29;8:471. doi: 10.1038/s41420-022-01257-1 (PMC9708830; doi:10.1038/s41420-022-01257-1)
Supplement: Supplementary file 2 — supplementary full length western blot [file 41420_2022_1257_MOESM2_ESM.pptx]

## Slide 1
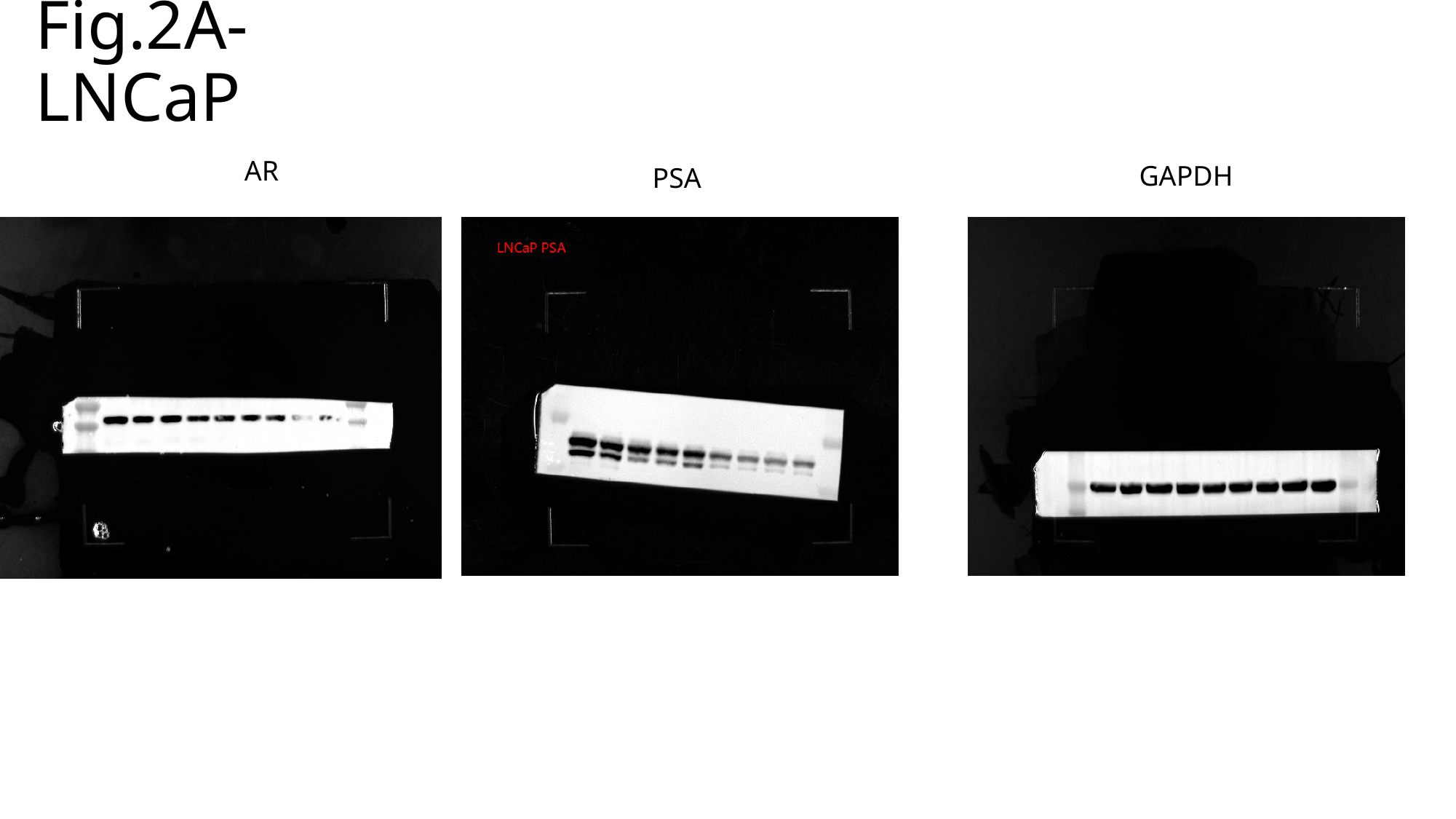

# Fig.2A-LNCaP
AR
GAPDH
PSA

## Slide 2
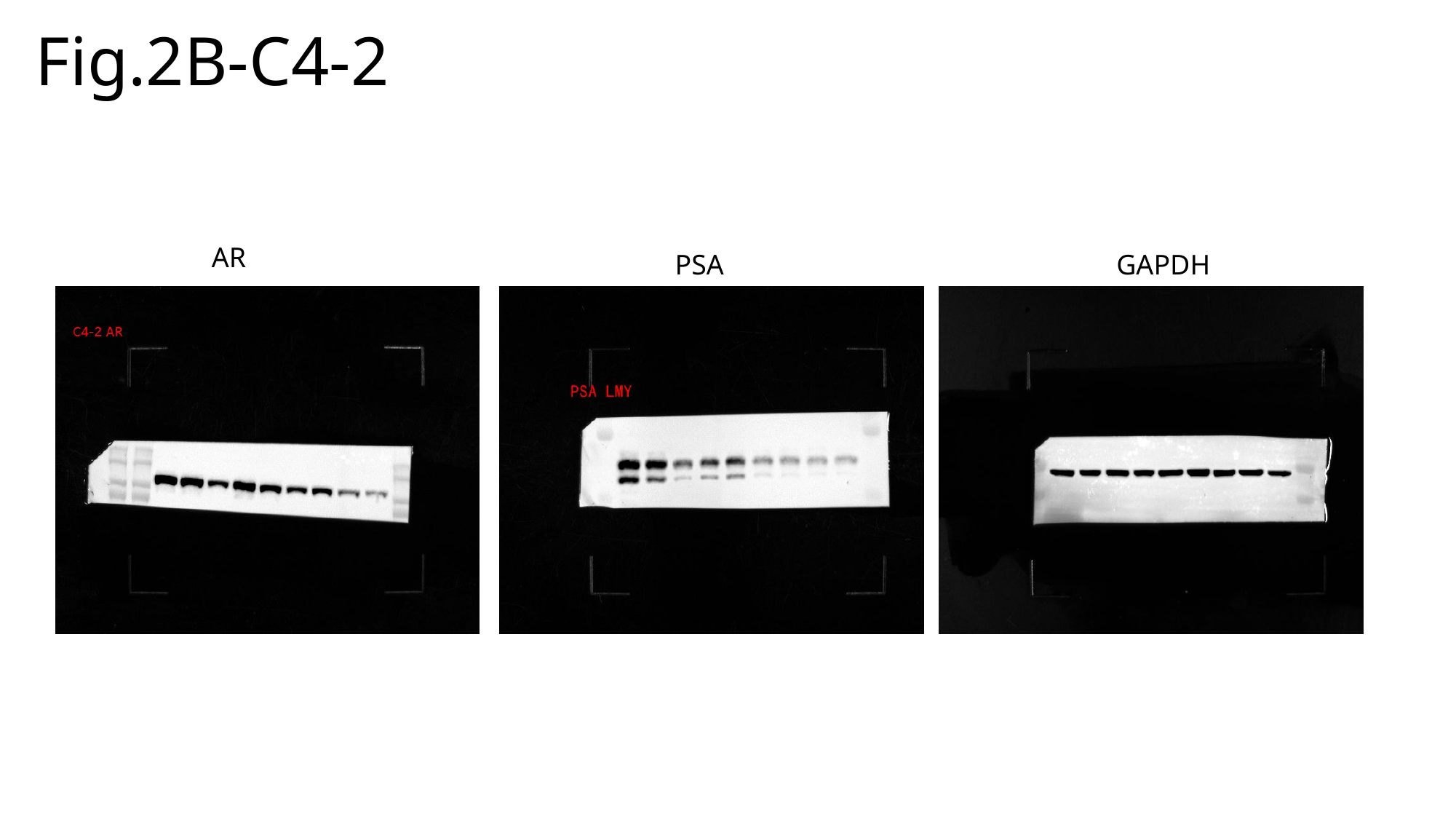

# Fig.2B-C4-2
AR
PSA
GAPDH

## Slide 3
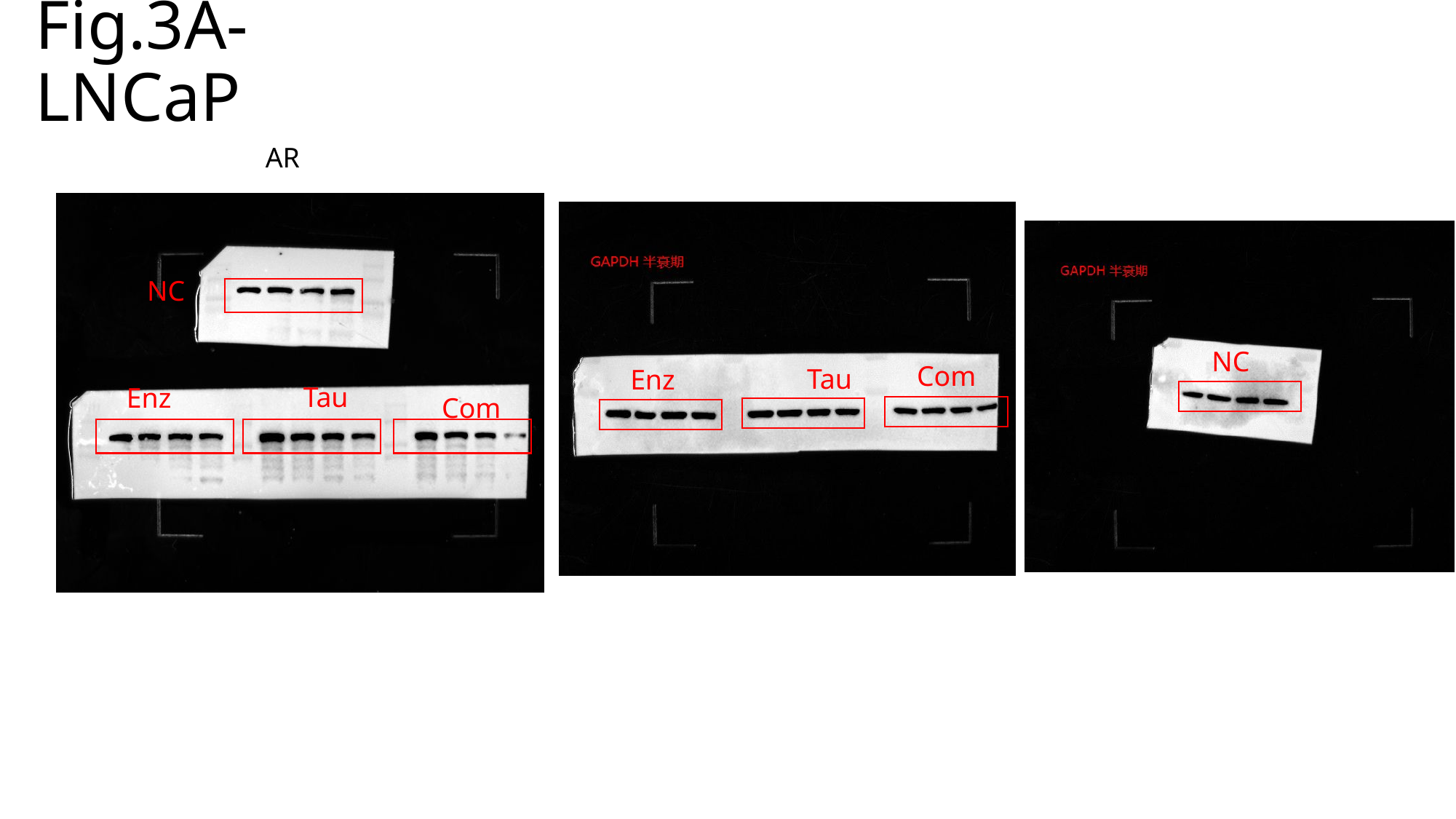

# Fig.3A-LNCaP
AR
NC
NC
Com
Tau
Enz
Tau
Enz
Com

## Slide 4
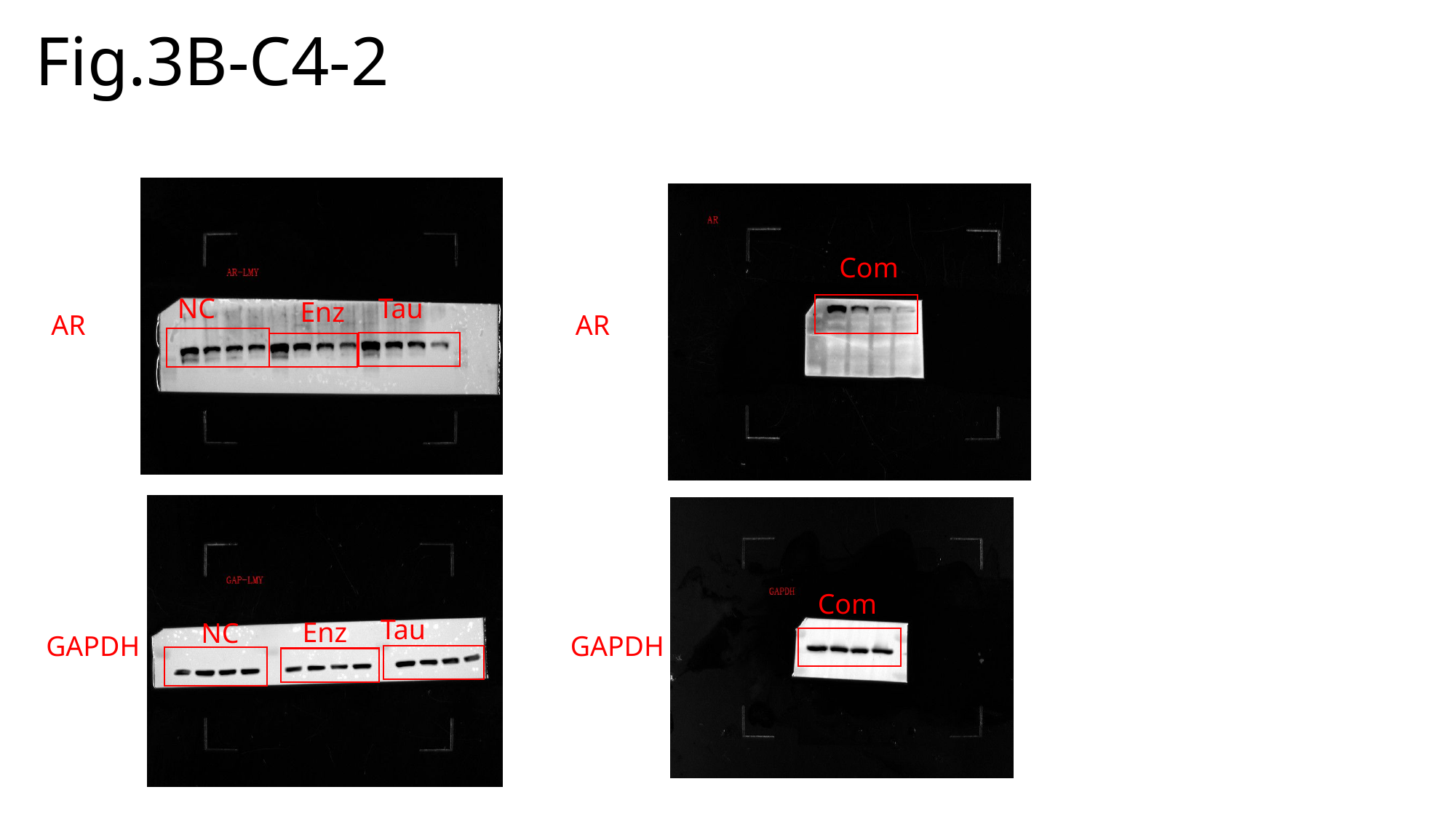

Fig.3B-C4-2
Com
NC
Tau
Enz
AR
AR
Com
Tau
Enz
NC
GAPDH
GAPDH

## Slide 5
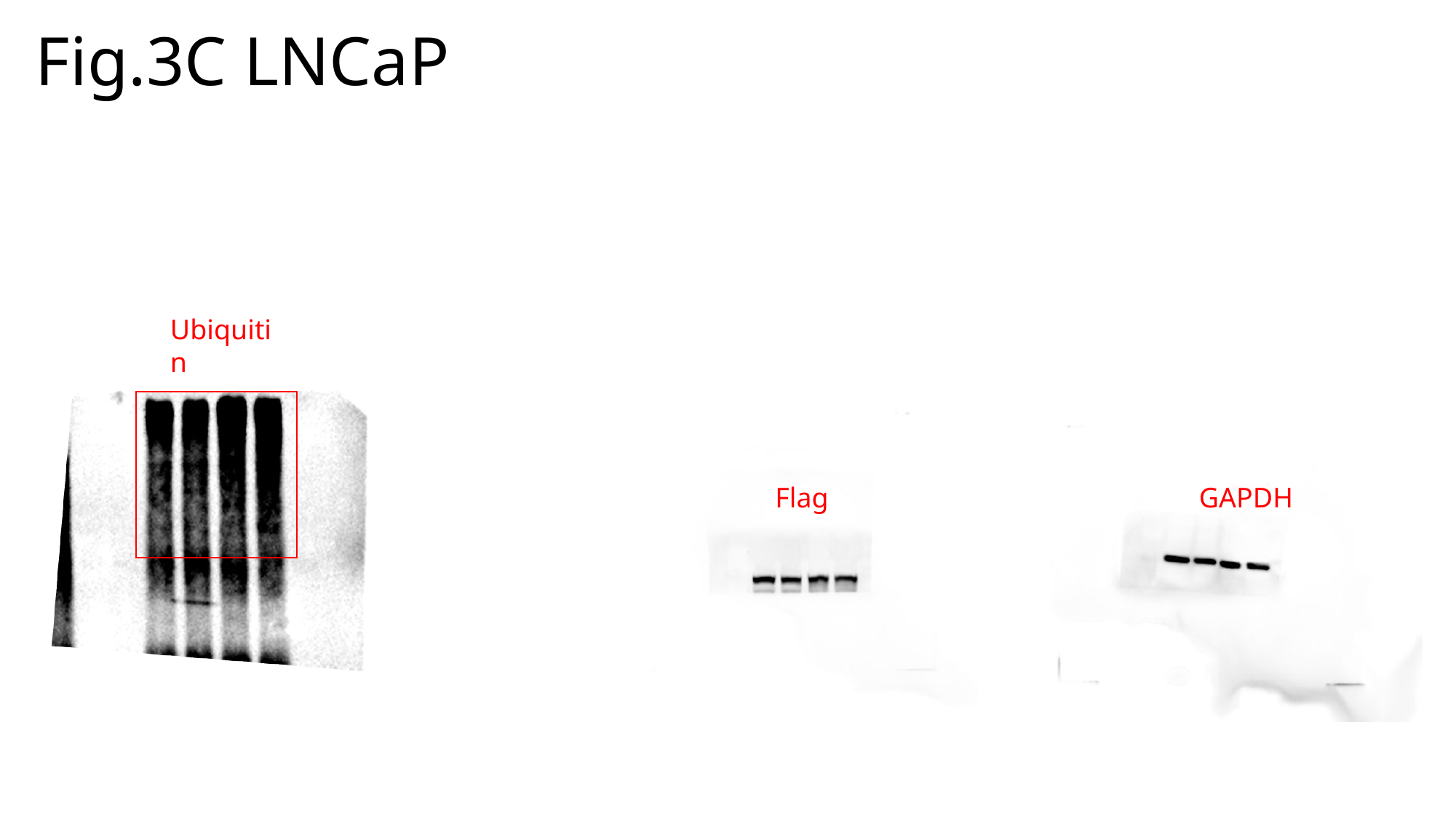

Fig.3C LNCaP
Ubiquitin
Flag
GAPDH

## Slide 6
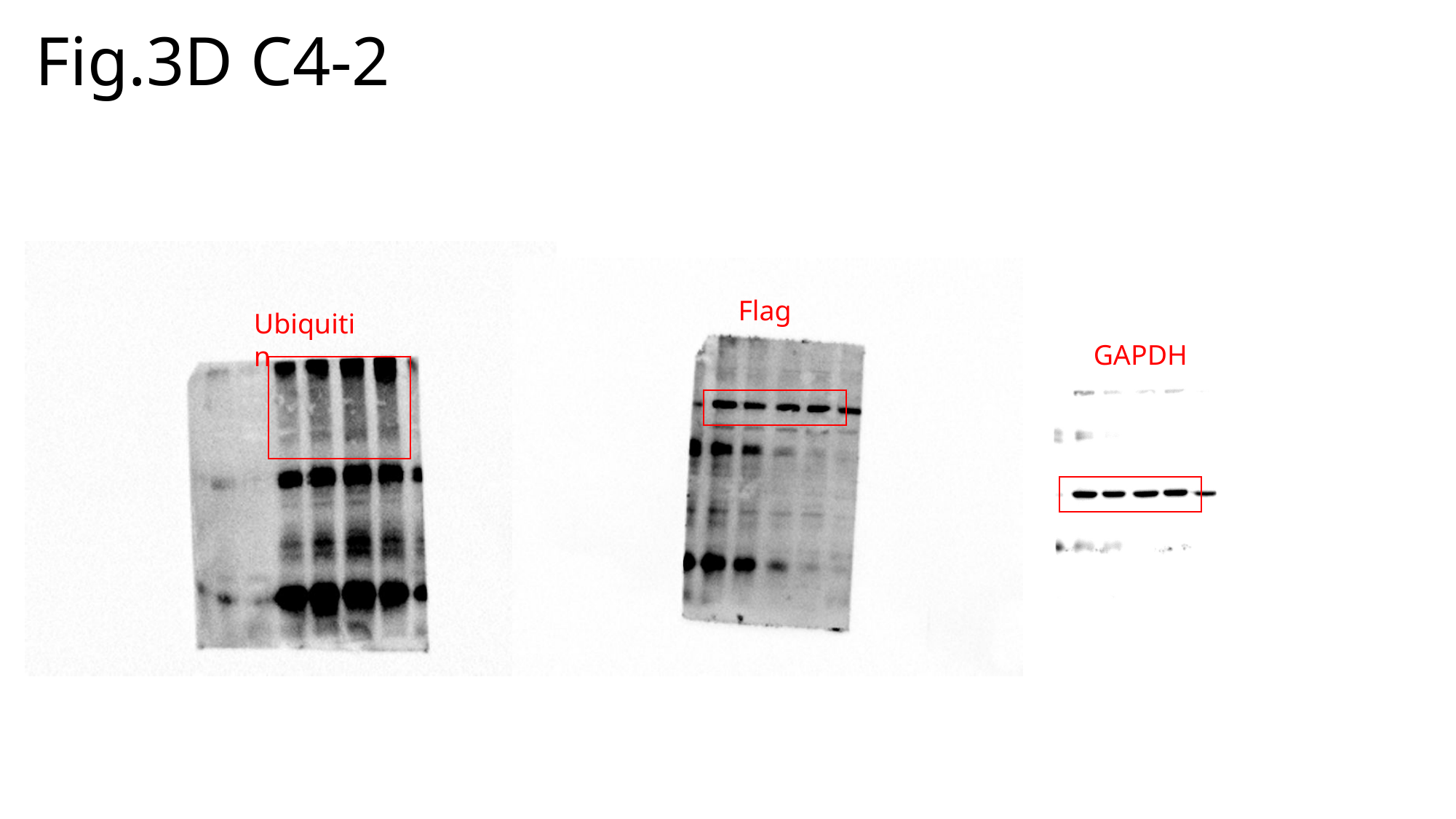

Fig.3D C4-2
Flag
Ubiquitin
GAPDH

## Slide 7
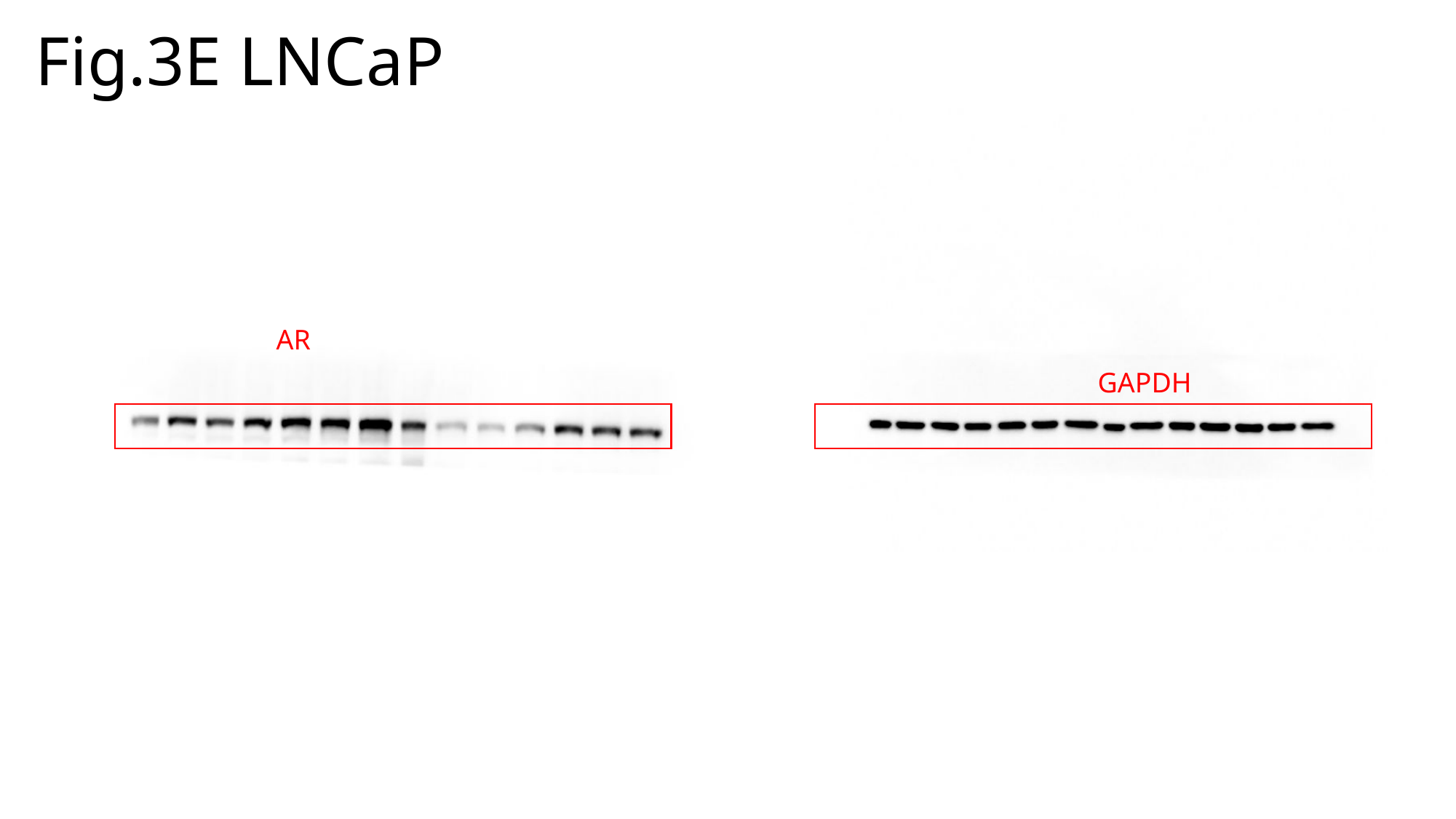

Fig.3E LNCaP
AR
GAPDH

## Slide 8
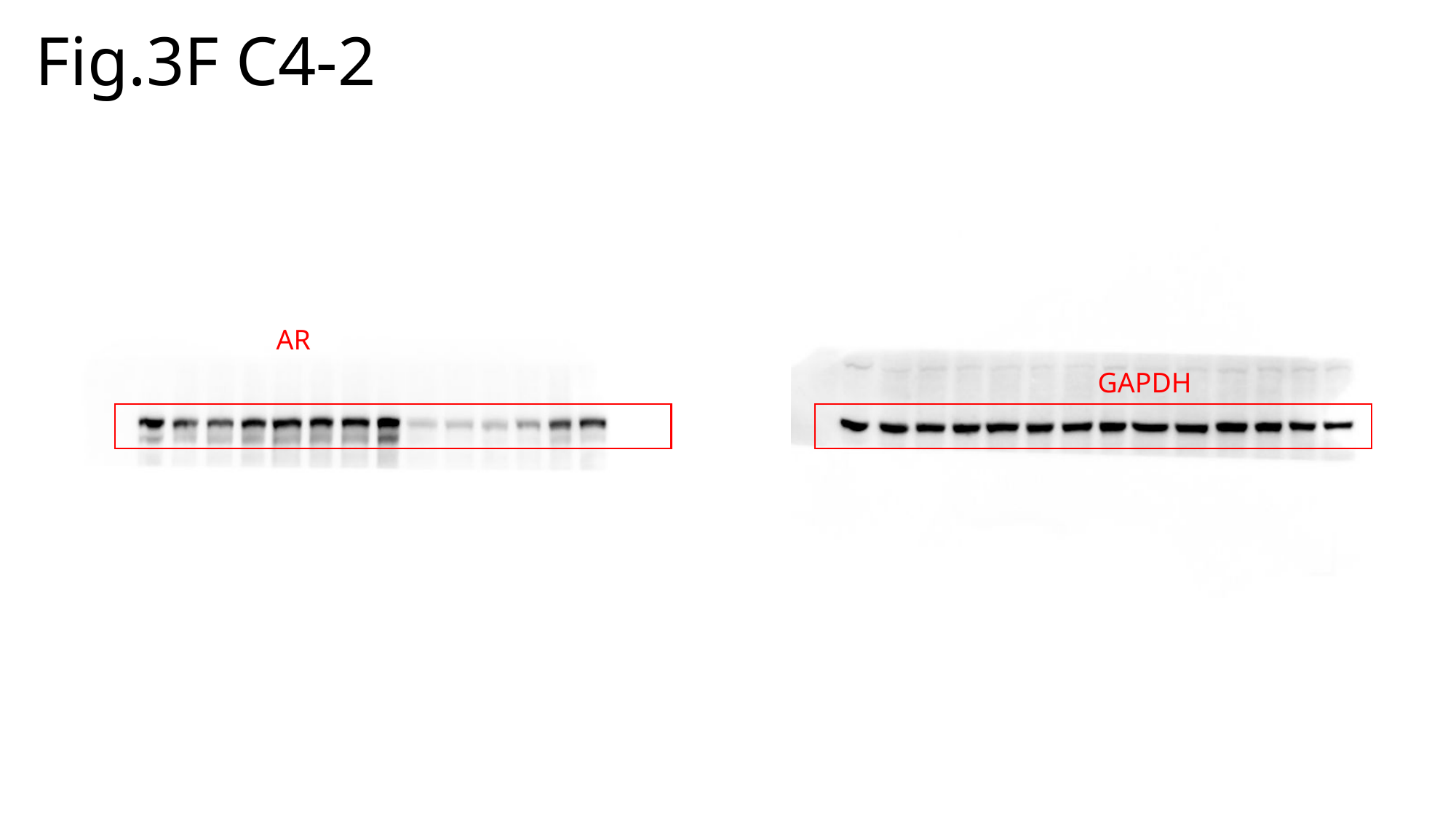

Fig.3F C4-2
AR
GAPDH

## Slide 9
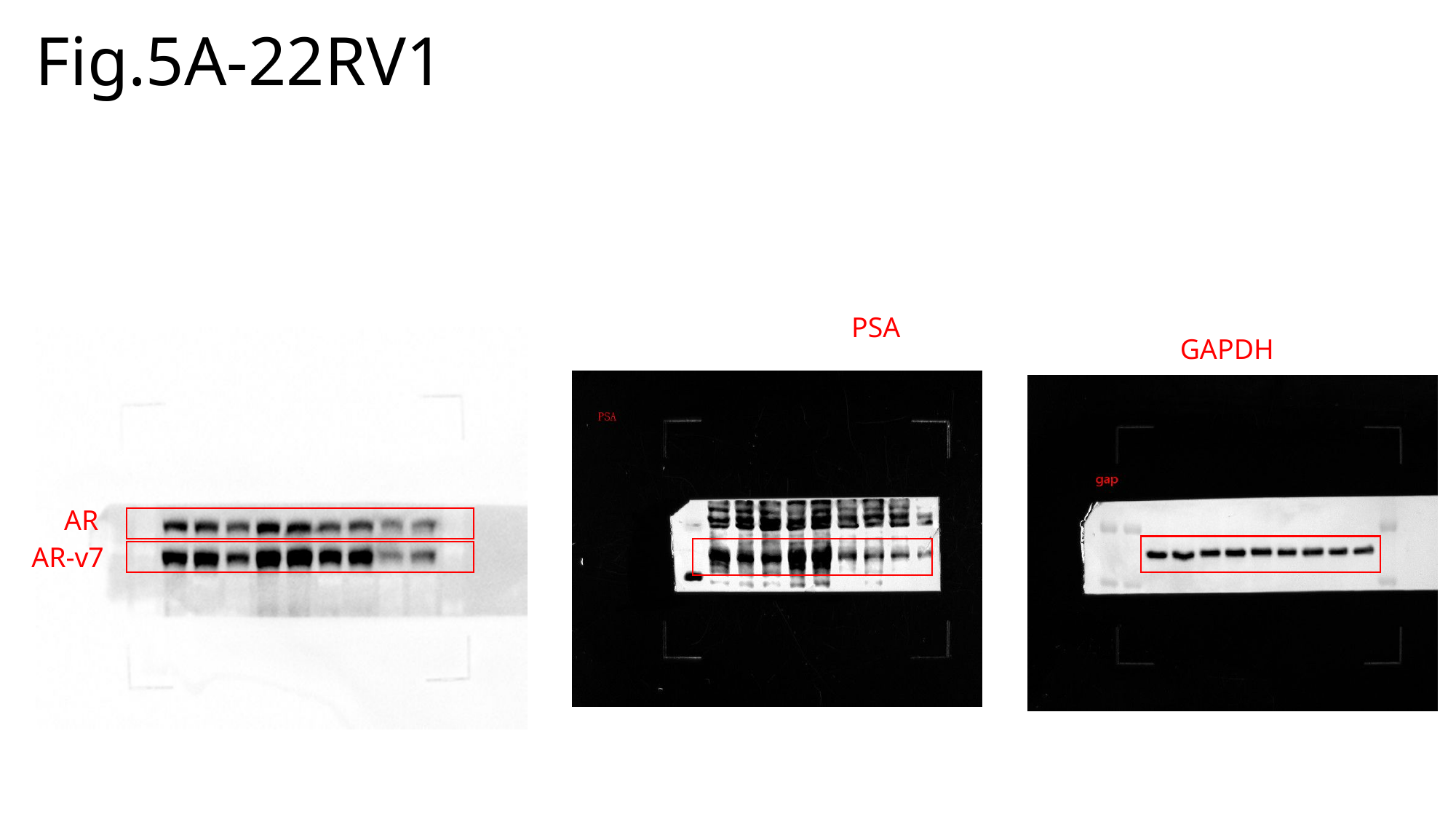

# Fig.5A-22RV1
PSA
GAPDH
AR
AR-v7

## Slide 10
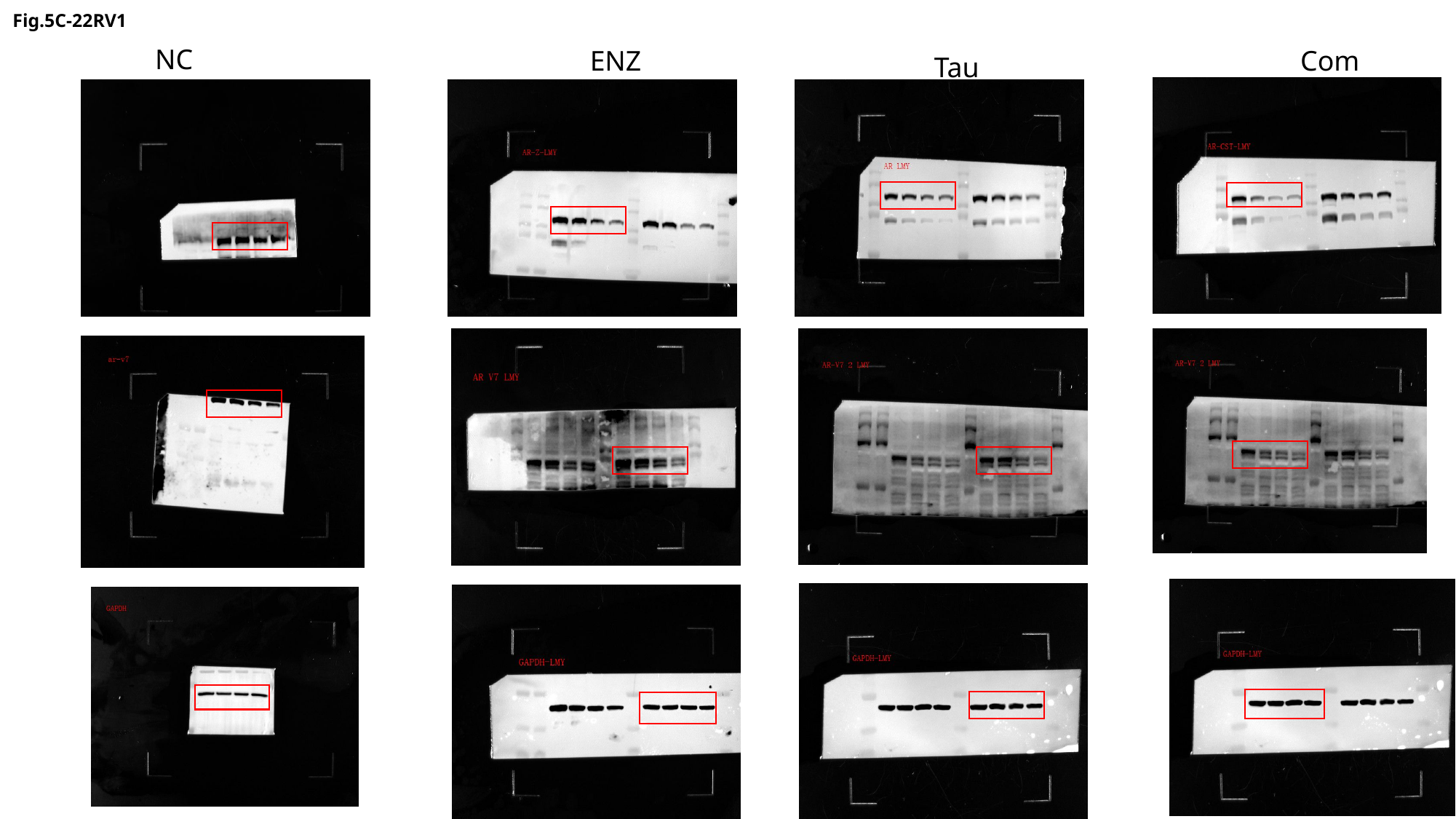

Fig.5C-22RV1
NC
ENZ
Com
Tau

## Slide 11
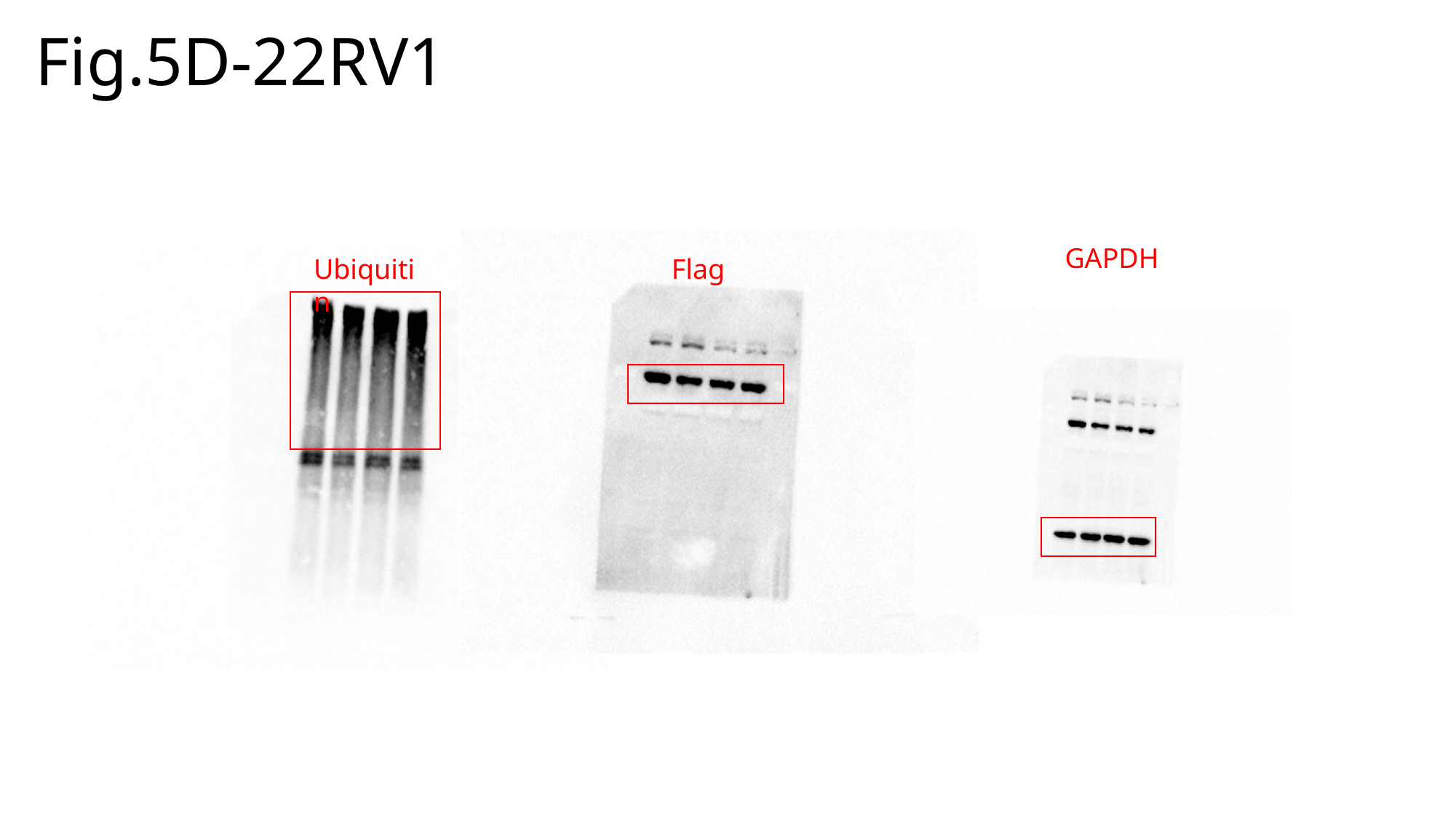

Fig.5D-22RV1
GAPDH
Ubiquitin
Flag

## Slide 12
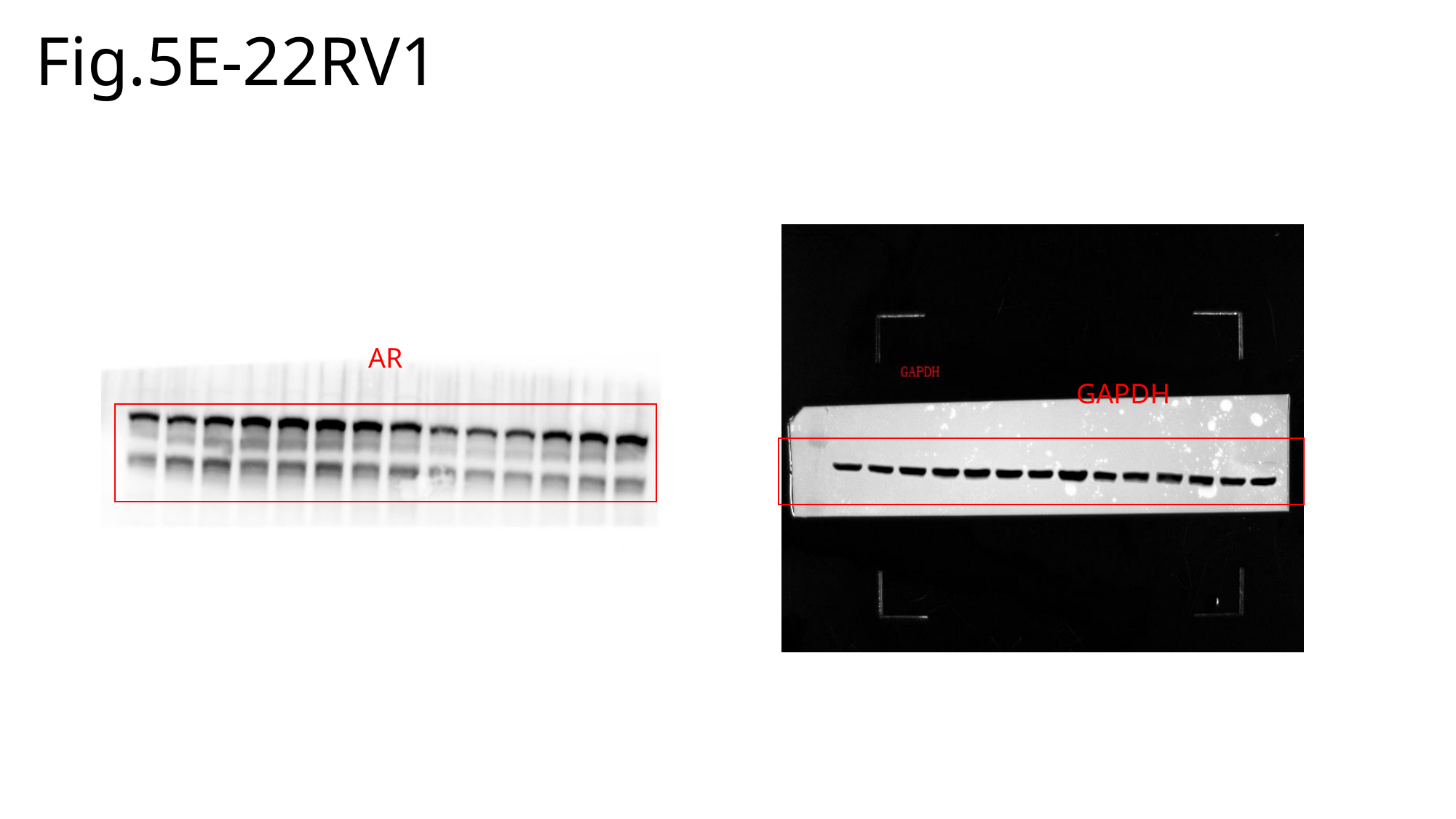

Fig.5E-22RV1
AR
GAPDH

## Slide 13
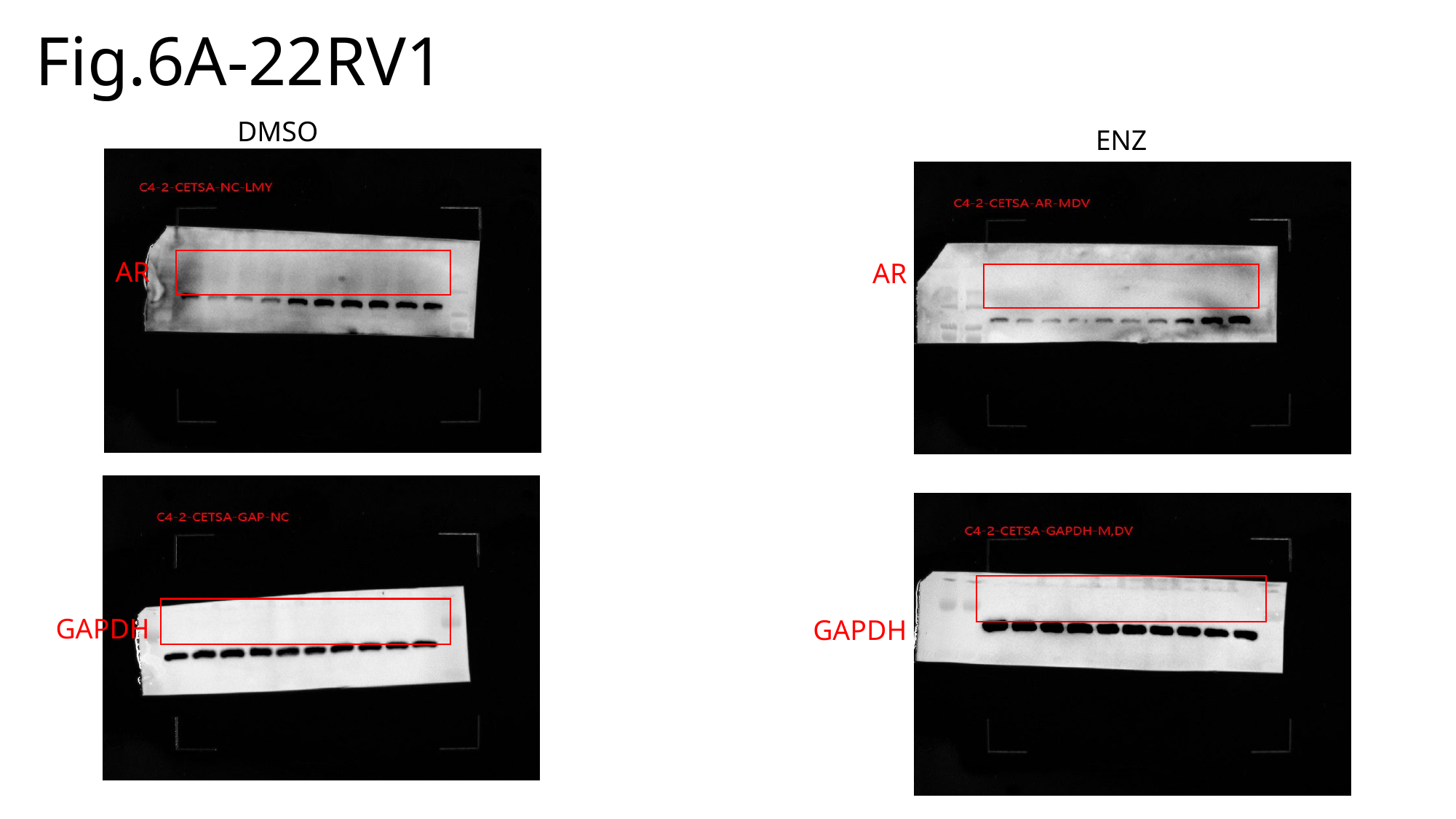

Fig.6A-22RV1
DMSO
ENZ
AR
AR
GAPDH
GAPDH

## Slide 14
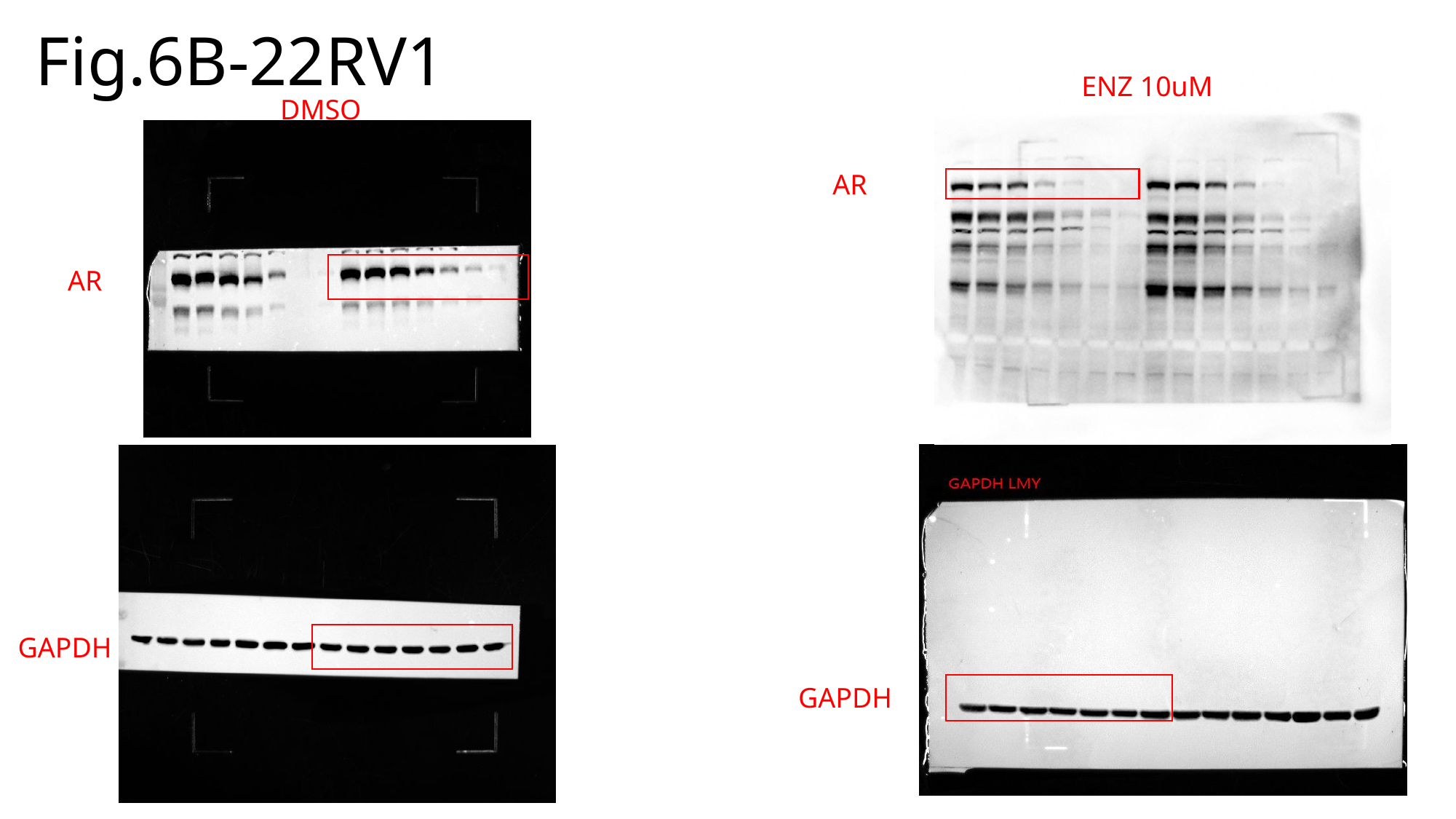

Fig.6B-22RV1
ENZ 10uM
DMSO
AR
AR
GAPDH
GAPDH

## Slide 15
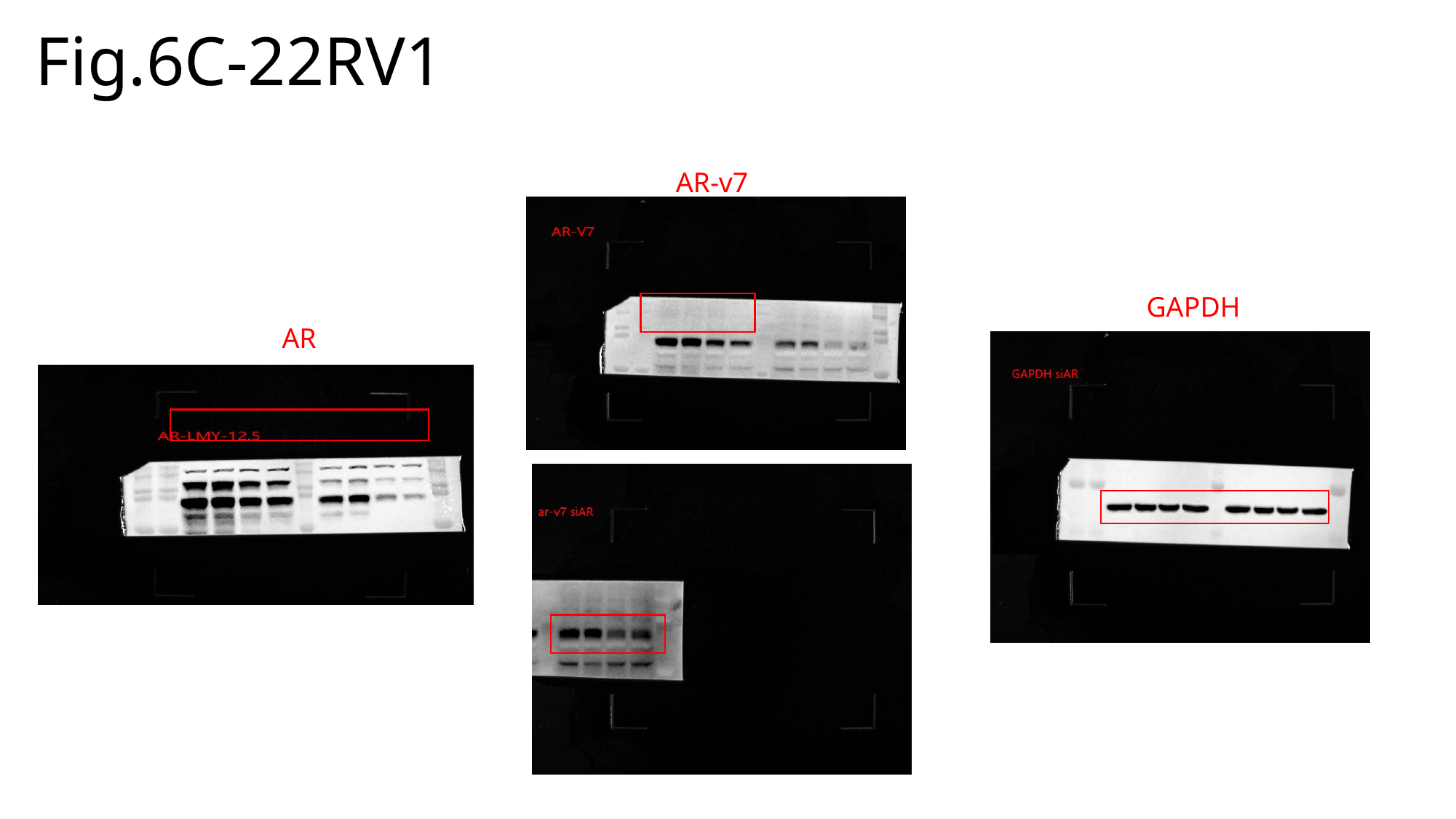

Fig.6C-22RV1
AR-v7
GAPDH
AR

## Slide 16
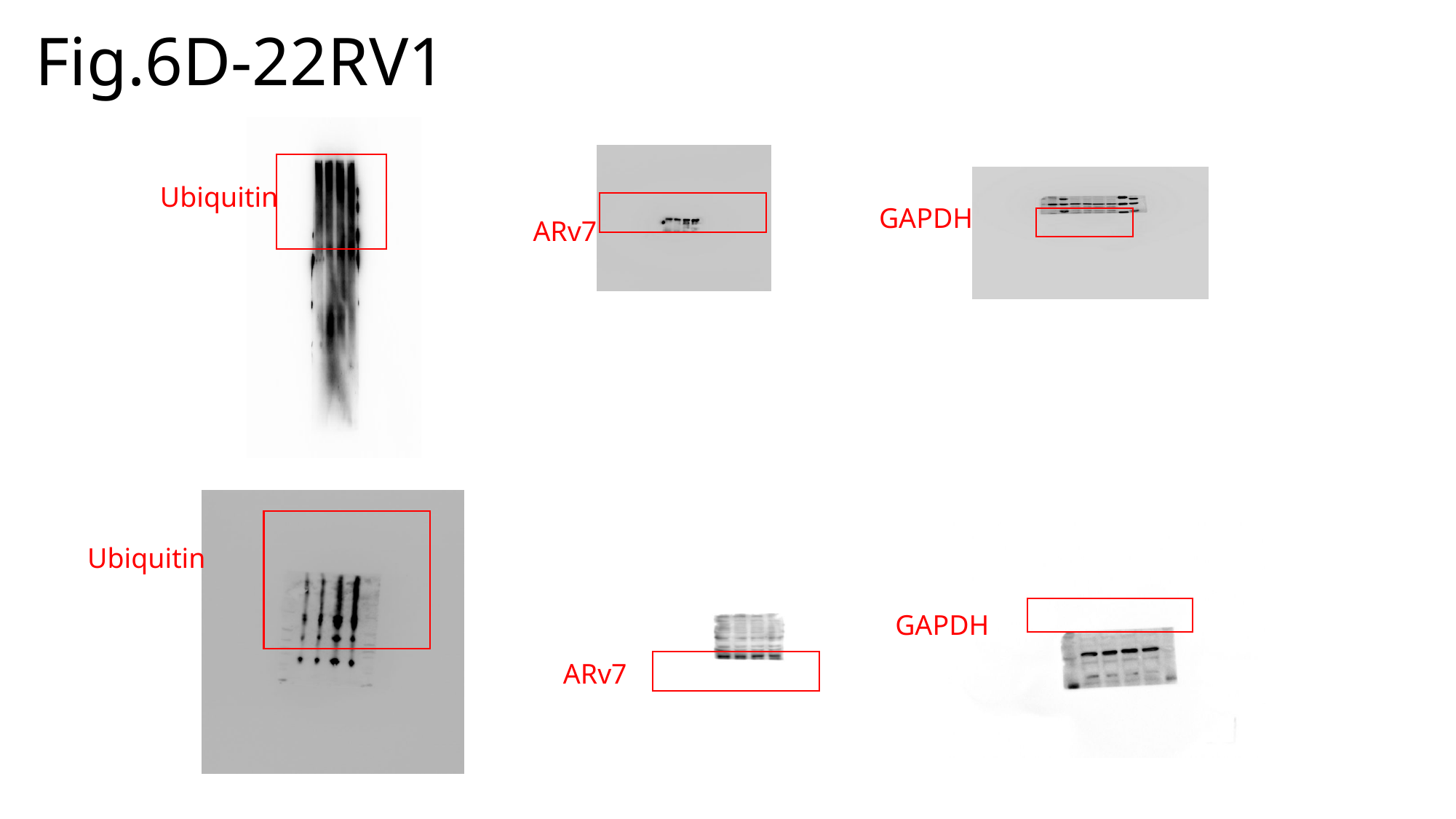

Fig.6D-22RV1
Ubiquitin
GAPDH
ARv7
Ubiquitin
GAPDH
ARv7
